# Physicochemical, Antioxidant, Sensory, and Starch Digestibility Properties of Steamed Bread Fortified with Tamarillo Powder

**DOI:** 10.3390/foods12122306

**Published:** 2023-06-07

**Authors:** Pei-Ci Syu, Qi-Fang Zhang, Sheng-Dun Lin

**Affiliations:** Department of Food Science and Technology, Hungkuang University, Taichung 433304, Taiwan; spc679163@gmail.com (P.-C.S.); 0712fa520@gmail.com (Q.-F.Z.)

**Keywords:** tamarillo, steamed bread, bioactive component, antioxidants, sensory evaluation, glycemic index

## Abstract

The effects of lyophilized tamarillo powder (TP) on the physicochemical, antioxidant, sensory, and starch digestibility characteristics of steamed breads were studied. The TP was used to substitute 5–20% of wheat flour to make steamed breads, assigned as T5, T10, T15, and T20, respectively. The results showed that TP is rich in dietary fiber (36.45%). Its extract is rich in bioactive components, including phenolic compounds (28.90 mg GAE/g extract), ascorbic acid (3.25 mg/g extract), total anthocyanins (316.35 μg C3GE/g extract), and total carotenoids (12.68 μg βCE/g extract) and has good antioxidant capacity. As the level of TP increased, the color of steamed breads became darker, redder, and yellower; the texture became harder, and the overall consumption preference decreased. However, their bioactive components content and antioxidant activity increased. The starch hydrolysis percentage of T5 (43.82%), T10 (41.57%), T15 (37.41%), and T20 (35.63%) at 180 min was significantly lower than that of the control (49.80%) (*p* < 0.05). The in vitro predicted glycemic index (80.02) of T20 was categorized as a medium-GI food when bread was used as the reference. On a nine-point hedonic test, control and T5 had the highest overall preference scores (7.1–7.4). The T20 supplemented with extra 15–20% water improved its volume and specific volume, and the overall preference scores (7.4–7.5) were not significantly different from the control (*p* > 0.05). Overall, a partial replacement of wheat flour with TP in steamed bread making could be developed as a new type of medium-GI value food containing more bioactive components and effective antioxidant capacity.

## 1. Introduction

Traditional steamed bread is one of the staple foods loved by Asians. Its basic ingredients are wheat flour, water, and yeast. After mixing the ingredients to form fermented dough, it is steamed for consumption. Compared with baked bread, the outer layer of traditional steamed bread is smooth and white, and because of the relatively low production temperature (<100 °C), the steamed bread contains fewer Maillard reaction products, such as acrylamide and furan [1]. However, several studies found that traditional steamed bread made from refined wheat flour, is a high-glycemic-index (GI, 88.1–98.3) product [2,3,4]. The intake of high-GI foods by the human body may cause a rapid increase in blood sugar levels and insulin secretion, which in turn increases the risk of cardiovascular disease [5]. Therefore, developing steamed bread with lower GI is beneficial for patients with diabetes and other diseases [6]. In view of this, many scholars have used plants, such as wholegrains and seeds, to improve the GI, physicochemical, antioxidant, and sensory qualities of the SB [7,8,9]. However, there is little information on the application of fruit to improve the GI of steamed bread.

Tamarillo (*Solanum betaceum* Cav.), also known as tree tomato, is a kind of Solanaceae fruit that grows in the subtropical climate of South America, especially the countries across the Andes Mountains. It is a subtropical fruit with unique color and flavor, rich in nutrients and bioactive components, including minerals; dietary fiber; non-starch polysaccharides; vitamins A, C, B_6_, E, and K, carotenoids; phenolic compounds; and anthocyanins [10,11,12,13,14,15]. Its potential health benefits comprise anti-obesity [16], anti-inflammatory [16], antibacterial [17], antioxidant [10,12,18,19], and anti-tumor [11,18] properties; inhibition of enzymes activity related to metabolic syndrome characteristics [19]; and a potential “multitarget” activity against Alzheimer’s disease [20]. Because of its bioactive ingredients and novel eating experience, tamarillo has been used in a variety of products, including salads, sauces, jellies, ice cream, juices, liqueurs, yogurt, and effervescent fruit tablets [13,14,21,22]. Therefore, the tamarillo is an important source that has the potential to be developed into healthy products.

Increasing intake of dietary fiber may be one way to lower postprandial blood glucose [7,23]. The content of dietary fiber and bioactive components in traditional SB is insufficient. Therefore, it would be beneficial to develop a new formulation of steamed bread containing tamarillo powder (TP). The purpose of this study was to measure the physicochemical quality characteristics of TP and wheat flour and then use 5%, 10%, 15%, and 20% (*w*/*w*) of TP instead of wheat flour to make steamed breads to evaluate the effect on the physicochemical, antioxidant, sensory, and starch digestibility properties of the steamed breads. Furthermore, the effect of various amounts of water on the quality characteristics of tamarillo steamed breads was also studied.

## 2. Materials and Methods

### 2.1. Materials

**Figure 1:**
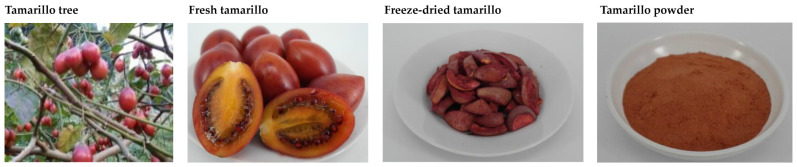
The appearance and color of tamarillo tree, fresh tamarillo, freeze-dried tamarillo, and tamarillo powder.

The red variety of tamarillo fruit (*Solanum betaceum* Cav.) (Figure 1) was provided from the farmer (Nantou, Taiwan) and harvested at 22–24 weeks after anthesis at 1100–1200 m sea level. The plant was 5 years old, and all harvested fruits are usually consumed. Then, the fruits were packed in cartons and were transported to Hungkuang University (about 2 h). Fresh fruits were washed with tap water, drained, and then cut into two pieces with a knife. The samples were subjected to vacuum freeze-drying using a freeze-dryer (FD-20L-6S, Kingmech Co., Ltd., New Taipei, Taiwan) with drying conditions of <0.2 mmHg, chamber temperature of 25 °C, and condenser temperature of −50 °C. Dried samples were ground using a mill (RT-30HS, Rong Tsong Precision Technology Co., Taichung, Taiwan) and screened through a 0.7 mm sieve. The dried powders were sealed in PET/Al/PE bags and then stored at −25 °C before use. Low-protein flour (LPF, protein, 8%) of soft white wheat and middle protein flour (MPF, protein, 11.6%) of hard red winter wheat from US were purchased from Chia Fha Enterprise Co., Ltd. (Taichung, Taiwan). Sugar (Taiwan Sugar Co., Ltd., Tainan, Taiwan), soybean powder (Mauhshii Co., Ltd., New Taipei, Taiwan), Lesaffer Saf-Instant yeast (red) (Lesaffer, Marcq-en-Baroeul, France), and shortening (Namchow Oil and Fat Co., Ltd., Taoyuan, Taiwan) were purchased from the local market.

Gallic acid, Folin–Ciocalteu phenol reagent, 1,1-diphenyl-2-picrylhydrazyl (DPPH), potassium ferricyanide, ferric chloride, pepsin, α-amylase (heat stable), α-amylase (from porcine pancreas, type VI-B), α-amylase (from human saliva, type XIII-A), 3,5-dinitrosalicylic acid, maltose, protease (from *Bacillus licheniformis*), amyloglucosidase, celite filter aid, tichloroacetic acid, ascorbic acid, and acetic acid were purchased from Sigma-Aldrich Chemical (St. Louis, MO, USA). Hexane was purchased from Tedia (Fairfield, OH, USA). Acetonitrile, methanol, and acetone were purchased from Avantor Performance Materials (Center Valley, PA, USA). Potassium chloride was purchased from Showa Chemical (Tokyo, Japan). Cupric sulfate, anhydrous sodium carbonate, and sodium hydroxide were purchased from Shimakyu’s Pure Chemicals (Osaka, Japan). Methyl red pure was purchased from Koch Light Research Laboratories (Gauteng, South Africa). Methylene blue and anhydrous sodium acetate were purchased from Katayama Chemical Industries (Osaka, Japan). Potassium sulfite was purchased from Nihon Shiyaku Reagent (Tokyo, Japan). Potassium sodium (+)-tartrate tetrahydrate was purchased from Wako Pure Chemical Industries (Osaka, Japan). Dialysis membrane (molecular weight cut-off, 6000–8000) was purchased from Membrane Filtration Products (Seguin, TX, USA). Sodium dihydrogen phosphate (monobasic), sodium phosphate (dibasic, 12 hydrates), sulfuric acid, and hydrochloric acid were purchased from Union Chemical (Hsinchu, Taiwan). Ethanol was purchased from Taiwan Tobacco & Liquor (Tainan, Taiwan).

### 2.2. Steamed Bread Making

For the control group of steamed bread making, the method and formula (Table 1) of traditional Chinese wheat food was followed, and the dough was prepared by a straight dough method [24]. The formula of the control steamed bread contained middle protein flour (160 g), low-protein flour (40 g), soybean powder (4 g), yeast (4 g), sugar (10 g), shortening (2 g), and water (110 g) on the basis of the formulation. The TP was used to substitute 5%, 10%, 15%, and 20% (*w*/*w*) of low-protein flour to make various steamed breads, designated as T5, T10, T15, and T20, respectively. First of all, TP was mixed with water, and all the ingredients (except the shorting) were poured into the mixing tank in a mixer (SC-20L, Sun-Mate Machinery Co., Ltd., New Taipei, Taiwan) at a low speed (146 rpm) for 2 min. Melted shortening was added into the paste and mixed using a mixer at low speed for 1 min and then turned to a high speed (585 rpm) for 4 min to complete the dough. The dough was divided into 110 g each, 3 parts in total. After folding 3 times by hand, it was shaped into a strip of 17 cm, the head and tail were cut off (16 cm left), and the dough was divided into 4 equal parts (25 g). The dough was placed into an incubator (SP-18S, Sun-Mate Machinery Co., Ltd., New Taipei, Taiwan) at 35 °C and 75% RH for 20 min. Conventional steaming was performed for 7 min in a steaming basket with a boiling wok underneath. Afterwards, the steamed breads were taken out of the basket, cooled to room temperature for 30 min and weighed. The steamed breads were packed in polyethylene bags before analyses of physicochemical and sensory characteristics. Twelve batches (12 steamed breads per batch) were manufactured for each formula. To determine the effect of adding extra water on the quality characteristics of T20, the formula of T20 contained middle protein flour (160 g), TP (40 g), soybean powder (4 g), yeast (4 g), sugar (10 g), shortening (2 g), and water (110 g) on the basis of the formulation (Table 1). The extra 5%, 10%, 15%, 20%, 25%, and 30% (*w*/*w*) amounts of water were on the basis of the amount of wheat flour in the control as 100%. The total amounts of water added to SB of T20-05, T20-10, T20-15, T20-20, T20-25, and T20-30 were 120 g, 130 g, 140 g, 150 g, 160 g, and 170 g, respectively.

### 2.3. Determination of Proximate Composition, Total Dietary Fiber, and Water Holding Capacity

The moisture, crude fat, crude protein, and crude ash of the samples were determined according to the method of American Association of Cereal Chemists [25]. The nitrogen conversion factor of wheat flour, TP, and steamed breads used for crude protein calculation was 5.7, 6.25, and 5.7, respectively. The total dietary fiber of the samples was analyzed according to the AOAC method 985.29 [26]. The carbohydrate content (g/100 g) and water holding capacity (g H_2_O absorbed/g sample) of the samples were measured according to the method of Mau et al. [27]. Each analysis was carried out in triplicate.

### 2.4. Determination of Physical Characteristics

The weight of steamed breads was measured using a scale. The maximum length, width, and height of the steamed breads were measured using a Vernier caliper (Mitutoyo, Kawasaki, Kanagawa, Japan). The spread ratio was calculated by dividing the maximum width with the maximum height of the steamed breads. The volume of the samples was determined by the rapeseed displacement method [28]. The specific volume was calculated by dividing the volume with the weight of the steamed breads. The water activity of the SB was determined using a water activity meter (LabSwift-aw, Novasina AG, Neuheimstrasse, Lachen, Switzerland). Each analysis was carried out with 10 replications.

The reflective color of the sample was measured using a color measurement spectrophotometer (ColorFlex-Diffuse, Hunter Associates Laboratory, Reston, VA, USA) set for CIE L*, a*, and b* values with a D65 illuminant at 10° [27]. The hue angle (h*), chroma (c*), and whiteness index (WI*) of the steamed breads were calculated as arctan (b*/a*) and (a*^2^ + b*^2^)^1/2^ and 100 − [(100 − L*)^2^ + a*^2^ + b*^2^]^1/2^, respectively. The total color difference (Δ*E*) between the sample and the control was calculated as Δ*E* = [(L*_c_ − L*_t_)^2^ + (a*_c_ − a*_t_)^2^ + (a*_c_ − a*_t_) ^2^]^1/2^, where L*, a*, and b* are the color coordinates in the control (c) and tamarillo bread (t), respectively. The results of each color property were averaged from 10 replications.

Texture profile analysis (TPA) of the midsection (2.5 cm × 2.5 cm × 2.5 cm) of the SB was measured using a texture analyzer (TA-XT2, 25 kg model; Stable Micro Systems, Surrey, UK) [27]. After the steamed breads were steamed and cooled to room temperature (25–27 °C), the upper and lower layers of the steamed breads were cut off, and then the crumb was cut into cubes (2.5 cm × 2.5 cm × 2.5 cm) for measurement. The recorded texture parameters were hardness, cohesiveness, springiness, gumminess, chewiness, and resilience. The results for each texture property were presented as the mean of 10 replicates.

### 2.5. Determination of Bioactive Components

Fifteen grams of the samples (wheat flour, TP, and lyophilized steamed bread powder) were extracted with 150 mL of 50% (*v*/*v*) aqueous ethanol in a water bath at 75 °C (150 rpm) for 30 min and then centrifuged at 4450× *g* for 30 min and filtered with Advantec No. 1 filter paper. The residue was re-extracted with one extra 150 mL portions of solvent as described. The combined ethanolic extracts were rotary evaporated (40 °C) and freeze-dried. Total phenols of lyophilized extracts were determined following the method of Mau et al. [27]. The total phenols content of the extracts was calculated according to the standard curve of gallic acid [760 nm absorbance = 0.0008 C_gallic acid_ (μg/mL) − 0.0123, R^2^ = 0.9995]. Results were expressed as milligram of gallic acid equivalent (GAE) per gram of lyophilized extract. Each analysis was carried out in triplicate.

Total anthocyanins of the extracts were measured by the pH-differential method [29]. Total anthocyanins of the extract were expressed as micrograms of cyanidin-3-glucoside equivalent (C3GE) per gram of lyophilized extract. Each analysis was carried out in triplicate.

Total carotenoids of the lyophilized extracts were analyzed following the method of Knockaert et al. [30] with minor modification. Briefly, each extract (500 mg) was extracted with 20 mL of extraction solvent (hexane: acetone: ethanol, 2:1:1, *v*/*v*) and 1 mL of 10% KOH solution in a water bath at 25 °C (125 rpm) for 1 h and then centrifuged at 4000× *g* for 5 min. The supernatant was diluted to 25 mL with the extraction solvent and then poured into a separatory funnel (protect from light). Next, 25 mL of deionized water was added, and the mixture was shaken vigorously to mix (to remove the cover to exhaust air) and left to stand for 30 min. The upper organic layer (yellow) was taken out, and the absorbance was measured at 450 nm. Analyses were performed three times. Total carotenoids of the extract were expressed as micrograms of β-carotene equivalent (βCE) per gram of lyophilized extract. Each analysis was carried out in triplicate. 

The ascorbic acid content in the extract was measured based on the method of Attia [31] with minor modification. The extract (100 mg) was dissolved in methanol (3 mL) using an ultrasonic bath with 40 kHz for 15 min, and the volume was adjusted to 5 mL with methanol and then centrifuged at 1463× *g* for 10 min (3000 rpm). The solution was then filtered using a PVDF syringe filter (13 mm × 0.45 μm) prior to injection onto a HPLC. The HPLC system consisted of a Hitachi L-2130 pump, a Hitachi 5430 diode array detector, and a Luna C18 (2) column (250 mm × 4.6 mm, 5 μm particle size; Phenomenex, Torrance, CA, USA). The column temperature was maintained at 25 °C. The mobile phase consisted of 0.1% acetic acid (in water) and acetonitrile (75:25, *v/v*) at a flow rate of 1.0 mL/min. The sample injection volume was 10 μL. The ascorbic acid in the samples was identified by comparing its relative retention time and UV spectra with the authentic compound. Ascorbic acid content was calculated on the basis of the calibration curves of the corresponding authentic compound. Each analysis was carried out in triplicate.

### 2.6. Determination of Antioxidant Properties

The scavenging ability of each extract (0–0.75 mg extract/mL for TP and wheat flour; 0–3.6 mg extract/mL for the steamed breads) for DPPH radicals was determined on the basis of the method of Shimada et al. [32]. Reducing power of each extract (0–3 mg extract/mL for TP and wheat flour; 0–15 mg extract/mL for the steamed breads) was determined according to the method of Oyaizu [33]. The EC_50_ value (mg extract/mL) is the effective concentration at which the DPPH radicals were scavenged by 50%, and the absorbance was 0.5 for reducing power. The EC_50_ values of TP and tamarillo steamed breads were obtained by the interpolation method of linear regression analysis, while the EC_50_ values of flour and control steamed bread were obtained by the extrapolation method of linear regression analysis.

### 2.7. Determination of Starch Hydrolysis and Predicted Glycemic Index (pGI)

Starch hydrolysis of the cooked steamed breads was measured according to the confinement (dialysis) system method of Germaine et al. [34]. Briefly, the cooked steamed breads (50–60 °C) containing 1 g of available carbohydrate were weighed into tubes containing 5 mL of 0.05 M sodium potassium phosphate buffer (pH 6.9) and were determined for the starch hydrolysis with the restricted system. At 0, 15, 30, 45, 60, 90, 120, and 180 min, aliquots (2 mL) of the dialysate were withdrawn, analyzed for reducing sugar content using the 3,5-dinitrosalicylic acid method, and compared with a maltose standard curve. The area under the curve (AUC) value for each starch hydrolysis curve was calculated using Sigmaplot 10.0 (Systat Software, San Jose, CA, USA). The hydrolysis index (HI) was obtained by dividing the area under the hydrolysis curve of the sample by the area of white bread. The pGI of starch hydrolysis value at 180 min was calculated using the formula (pGI = 39.71 + (0.549 × HI)) developed by Goñi et al. [35]. Each analysis was carried out in triplicate.

### 2.8. Sensory Evaluation

The SB samples (50–60 °C) were subjected to sensory evaluation using the hedonic test. Ninety untrained consumers (32 males and 58 females, aged 18–60 years) who were interested in the SB samples were recruited from the students and staff at Hungkuang University. Each SB sample was placed on a white plate and identified with a random three-digit number, then presented to consumers in a balanced and random order. Consumers were asked to clean their mouths with warm water between sampling sessions to reduce the effect of sample carryover. The sensory quality characteristics of the SB samples was assessed using a nine-point hedonic scale questionnaire, including crust color, crumb color, sweetness, aroma, flavor, texture, and overall preference (1 = extremely disliked, 5 = neither liked nor disliked, 9 = extremely liked).

### 2.9. Statistical Analysis

With the exception of weight (*n* = 10), dimension (*n* = 10), texture profile analysis (*n* = 10), color value (*n* = 10), and hedonic testing (*n* = 90), each measurement was performed in triplicate. The experimental data were subjected to analysis of variance using a statistical analysis system (SAS Institute, Cary, NC, USA). Duncan’s multiple range tests were used to determine the difference among means at the 0.05 level.

## 3. Results and Discussion

### 3.1. Physicochemical Qualities of Tamarillo Powder and Wheat Flour

The TP used in this study was rich in total dietary fiber (TDF) and crude ash but lower in moisture, carbohydrate, and water activity than wheat flour (Table 2). The presence of a large amount of ash correlated with the quantity of minerals present in the TP [11]. TP has a higher water holding capacity (WHC) than wheat flour. Robertson and Eastwood [36] reported dietary fiber has water holding capacity. Therefore, this result should be attributed to the abundance of TDF in TP.

The a*, b*, and c* values of TP were significantly higher than those of wheat flour, but the L* value showed the reverse (Table 2), which means that the color of TP is redder, yellower, darker, and has higher color intensity than that of wheat flour. The 0°, 60°, 120°, 180°, 240°, and 300° of the h* are red, yellow, green, cyan, blue, and magenta, respectively [37]. The h* value of TP is 48.83° (Table 2), which is between red and yellow, consistent with the color of tamarillo powder in Figure 1. Interestingly, wheat flour is a white powder and cannot be suitably expressed by h*. Instead, it should be expressed by whiteness index. After calculation, the whiteness index of middle protein flour and low-protein flour were 88.97 and 90.57, respectively. As for TP, it is not suitable to be represented by whiteness index. The Δ*E* value of TP and wheat flour was >3. Song et al. [38] reported that the Δ*E* value can be explained as follows: Δ*E* < 1, the color difference was not obvious to the human eye; 1 < Δ*E* < 3, the color has no obvious difference from the human eye; Δ*E* > 3, the color difference was obvious to the human eye. The Δ*E* value of middle protein flour and low-protein flour was 1.24, which means that there was no obvious difference in the color of the two flours when viewed by the human eye.

TP and wheat flour were extracted with 50% aqueous ethanol. The extraction yield of the freeze-dried extract of TP was significantly higher than that of wheat flour extract (Table 3). Compared with wheat flour, this showed that TP has many components soluble in 50% ethanol. As expected, the TP in this study was rich in total phenols, ascorbic acid, total anthocyanins, and total carotenoids (Table 3). This result was similar to other studies [10,11,12,13,14,15]. Free radicals have been reported to be involved in the progression of various human diseases such as diabetes and atherosclerosis [39]. Naturally occurring antioxidants have been reported to delay the progression of many chronic human diseases by scavenging free radicals [40]. Studies have pointed out that TP has good antioxidant properties [10,12,18,19]. This should be related to the phenolic compounds and ascorbic acid [41], anthocyanins [42], and carotenoids [43] having good antioxidant properties. The results of the antioxidant capacity of the extracts in this study were expressed as EC_50_ values, which were negatively correlated with the antioxidant capacity. The scavenging ability on DPPH radicals and reducing power of TP extract were significantly higher than those of wheat flour extract, which should be related to the fact that TP was rich in phenolic compounds, ascorbic acid, anthocyanins, and carotenoids (Table 3), especially phenolic compounds and ascorbic acid [41]. Previous studies showed that the EC_50_ values of ascorbic acid for DPPH radical scavenging ability and reducing power were 15.49 μg/mL and 19.92 μg/mL, respectively [41]. Clearly, TP is a rich source of dietary fiber and bioactive components, and substituting TP for wheat flour in traditional steamed bread formula would result in a functional product.

### 3.2. Physical Characteristics of the Steamed Bread

There was no significant difference in the weight of steamed breads (Table 4). However, replacing wheat flour with TP will reduce the dimensions of steamed breads, including length, width, height, spread ratio, volume, and specific volume. The volume (mL) of the steamed bread samples decreased significantly from 78.5 (control) to 44.0 (T20) as the level of TP in the formulae increased. The decreased volume (%) of T5, T10, T15, and T20 was 1.91%, 11.5%, 26.8%, and 43.95%, respectively, compared to the control. This could be attributed to the gluten dilution effect, since TP does not contain gluten-type proteins. The gliadins and glutenins in flour are the gluten-forming proteins that provide the desired texture and volume of bread [44] In addition, the dietary fiber disrupted the gluten matrix of wheat flour in the steamed bread, which reduces the gas holding capacity of bread and the mechanical strength of dough during proofing, and steaming is also one of the reasons [45,46]. This change due to gluten dilution has been previously observed in steamed breads fortified with other functional ingredients, such as wholegrain flour, quinoa flour, high-amylose corn starch, and purple sweet potato flour [7,46,47,48].

**Figure 2:**
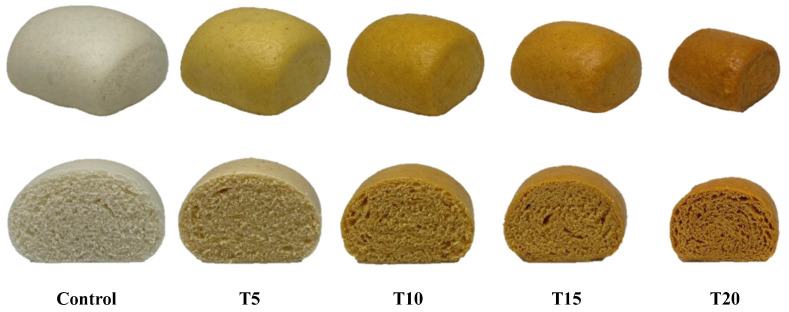
The appearance and color of tamarillo steamed breads.

Color is one of the important quality characteristics of food. The significant difference on the crust and crumb color of the steamed breads can be seen in Figure 2. The color of the steamed breads was greatly affected by the replacement of flour with TP (Table 4). For crust and crumb color, L* values decreased with the increase in TP levels, but the a*, b*, and c* values showed the reverse. This result indicated that the color of steamed breads was darker, redder, yellower, and had higher color intensity with the increase in TP levels. The color change of the steamed breads should be related to the pigments in TP, including anthocyanins and carotenoids. From the h* results of T5–T20, it was observed that the progression from yellow to red in crust color (77.51–60.91°) and crumb color (79.39–65.32°) correlated with increased TP substitution (Table 4). Compared with the control, the Δ*E* of the crust and crumb color of the tamarillo steamed bread samples ranged from 17.12 to 35.77 and 14.12 to 30.33, respectively (Table 4). This result indicated that the color difference between T5–T20 and control could be obviously distinguished by the human eye [38]. The crust color (Δ*E*, 1.88) of T15 and T20 and the crumb color (Δ*E*, 2.67) of T10 and T15 were difficult to distinguish. The Δ*E* values of other tamarillo steamed breads were greater than 3, which should be easy to distinguish with the naked eye [38].

Hardness is an indicator commonly used to evaluate the texture quality of food. In texture profile analysis, the order of hardness of the steamed breads was T20 > T15 > T10 > T5 > control, indicating that the samples became firmer with the increase in TP levels in the mixed formulae (Table 4). In this study, there was no significant difference in the weight of any of the steamed breads. Therefore, the increase in hardness is mainly related to the volume or specific volume of these samples. The increase in firmness of the food might be attributed to the density of the tested sample, which is inversely proportional to its specific volume. The internal resistance of the steamed breads’ structure can be expressed by cohesiveness; that is, the steamed breads with high cohesiveness form clumps rather than disintegrating during chewing [49]. The cohesiveness of the steamed breads decreased with the increase in TP level. Springiness is measured by the degree of recovery between the first and second compressions. Resilience is the percentage at which energy can be recovered when the first compression is released. The results of TPA showed that the springiness and resilience of steamed breads were in the order control > T5 > T10 > T15 > T20. This might be due to the higher proportion of TP replacing wheat flour in the formula of steamed breads, resulting in lower gluten content, smaller volume, and higher density. The energy required to break down food for swallowing can be expressed in terms of chewiness. Chewiness is calculated by multiplying gumminess by springiness, while gumminess is calculated by multiplying hardness by cohesiveness. Gumminess and chewiness of the SB increased with the increase in TP levels. Overall, as the level of TP in the formulae increased, the hardness, gumminess, and chewiness of the steamed breads increased, while their cohesiveness, springiness, and resilience decreased. Changes in the texture data of steamed breads could be used as quality indicators for subsequent consumer evaluation results.

### 3.3. Proximate Composition, Total Dietary Fiber, and Water Activity of the Steamed Bread

The crude ash, crude protein, and TDF contents of the steamed breads increased with the increase in levels of added TP (Table 5). This might be attributed to the fact that ash, protein, and TDF content of TP were higher than those of low-protein flour (Table 2). The moisture content of control and T5 did not differ significantly but was higher than that of T10, T15, and T20 (Table 5). This might be attributed to the fact that moisture content of low-protein flour was higher than that of TP (Table 2). The water activity of the steamed breads also decreased with the increase in TP levels, which could be attributed to the fact that TP has a higher WHC than low-protein flour (Table 2). The American Diabetes Association recommends that diabetes mellitus patients or the general population should consume 20 to 35 g of fiber from raw vegetables and unprocessed grains (or about 14 g of fiber per 1000 kcal ingested) per day [50]. According to the TDF data in Table 5, the tamarillo steamed breads in this study should have potential, especially T20. However, further research is required.

### 3.4. Bioactive Components and Antioxidant Activity of the Steamed Bread Extract

The steamed bread used 50% aqueous ethanol as the extraction solvent, and the extraction yields (15.72–25.43%) increased as the amount of low-protein flour replaced by TP increased (Table 6). The total phenols, ascorbic acid, total anthocyanins, and total carotenoids content of the lyophilized extracts also increased significantly with the increase in TP levels. This should be attributed to the higher bioactive content of the TP than that of low-protein flour (Table 3). When the extraction yields of the steamed bread were taken into consideration, the contents of total phenols, ascorbic acid, total anthocyanins, and total carotenoids of the steamed bread also increased significantly with the increase in TP levels. The DPPH radical scavenging efficiency and reducing ability of the steamed bread extracts were in the order of T20 > T15 > T10 > T5 > control (Table 6). The results showed that the substitution of TP for low-protein flour enhanced the antioxidant activity of the steamed bread. The improved antioxidant properties of the steamed bread could be attributed to the bioactive components of TP, including phenolic compounds, ascorbic acid, and carotenoids. Therefore, compared with traditional steamed bread, tamarillo steamed bread retained a large number of bioactive components and stronger antioxidant properties after cooking, which is beneficial to consumers and provides them with so-called physiological properties.

### 3.5. Starch Hydrolysis and pGI of the SB

**Figure 3:**
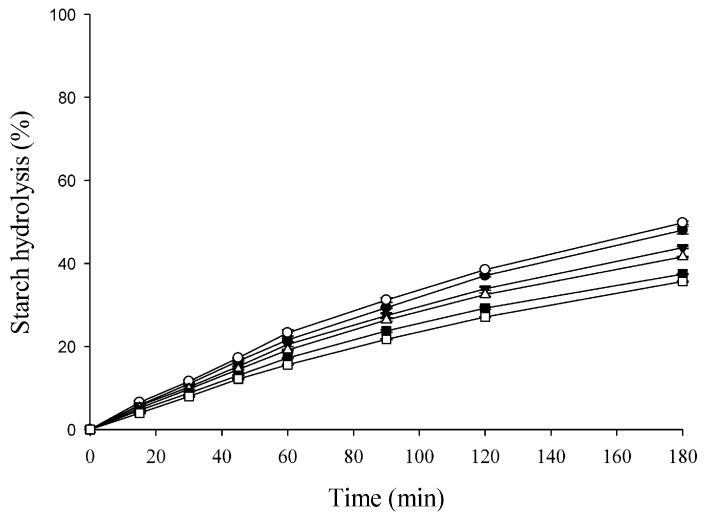
Starch hydrolysis (%) of white bread and tamarillo steamed breads. Each value is expressed as mean ± standard deviation (*n* = 3). White bread (●), control (○), T5 (▼), T10 (△), T15 (■), and T20 (□).

The in vivo measurement of GI requires the use of human subjects to assess glycemic responses 2 h after food intake; thus, it is resource-intensive and relatively time-consuming. The in vitro enzymatic assays to estimate the possible rates of starch digestion and glucose absorption in the small intestine are a faster and more cost-effective method than in vivo GI measurements [34,35]. The sensitivity of starch to the action of digestive enzymes is an important factor in both in vivo and in vitro responses; thus, the in vitro method allows the estimation of biological responses. It is particularly suitable for screening starch-rich products for GI during product development before selection for in vivo GI testing [35]. Therefore, the in vitro starch hydrolysis was used to estimate the pGI of the steamed breads in this study. As shown in Figure 3, when using white bread as a reference, the percentage of starch hydrolysis reached 48.01% at 180 min in the restricted system. The results were consistent with those of Granfeldt et al. [51] (nearly 50%), Germaine et al. [34] (about 46%), and Lai et al. [52] (about 42%) when using a restricted system. If an unrestricted system was used, the percentage of starch hydrolysis of white bread was approximately 70–80% after 180 min of incubation [34,35]. The starch hydrolysis curves of the tested steamed breads using the dialysis system followed a linear model. At the end of the 180 min incubation period, the percentage of starch hydrolysis was significantly lower for T5 (43.82%), T10 (41.57%), T15 (37.41%), and T20 (35.63%) than for the control (49.80%). The results suggest that the steamed breads made with TP instead of low-protein flour may be more beneficial to the health of general consumers and type 2 diabetes patients. The calculated pGI values for the steamed breads estimated from the ratio between the area under the hydrolysis curve for a given steamed breads and white bread are given in Table 7. The pGI values of control, T5, T10, T15, and T20 ranged from 97.28 to 80.02 when using white bread as the reference food. Traditional steamed bread is generally classified as a high-GI food [2,3,4]. Among the tested steamed breads, T20 had the lowest pGI value and was a medium-GI food (Table 7), which could be attributed to it having the highest content of TDF and bioactive components (Table 2 and Table 3).

### 3.6. Sensory Evaluation

**Figure 4:**
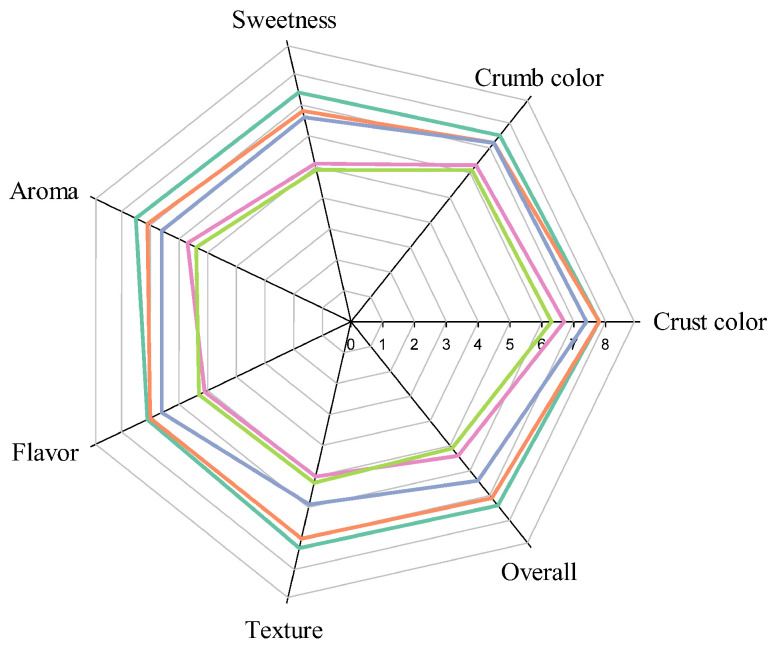
The radar plot of hedonic quality characteristics results of tamarillo steamed breads. Each value is expressed as mean ± standard deviation (*n* = 90). Control (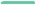), T5 (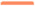), T10 (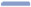), T15 (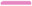), T20 (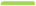). Values of each hedonic scale were averaged from 90 replicates. Nine-point hedonic scale with 1, 5, and 9 representing extremely disliked, neither liked nor disliked, and extremely liked, respectively.

The nine-point hedonic scale could be used as a measure of preference for products and provided reliable and valid results [53]. No significant differences were observed with regard to the color of the crust and crumb, sweetness, aroma, flavor, texture, and overall preference scores between the control and T5 (Figure 4). Results for all hedonic quality characteristics scores were 6.8–7.8 for control and T5, indicating that both samples were moderately to very much liked by consumption. The sweetness (5.1–6.6), aroma (5.3–6.6), flavor (5.1–6.6), texture (5.0–5.9), and comprehensive (5.1–6.4) scores of T10, T15, and T20 were significantly lower than those of the control. Consumption preferences ranged from “neither liked nor disliked” to “moderately liked”, although the hedonic quality characteristics scores of T15 and T20 were lower than those of other steamed breads. From the opinions of consumers in the questionnaire, if the moistness and texture of T15 and T20 could be improved, the consumers would also like T15 and T20, which are rich in bioactive components, TDF, and antioxidant capacity.

### 3.7. Effect of Added Water Amount on the Physical and Sensory Properties of T20

**Figure 5:**
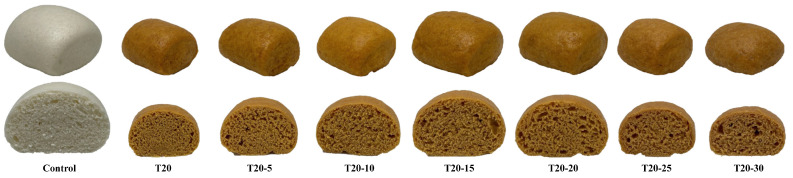
The appearance and color of control and T20 steamed breads with various amounts of water.

Since T20 contains more bioactive components, TDF, and better antioxidant properties than T15, it had the lowest pGI value. Consequently, T20 was used as the test sample in this study to investigate the effects of various water additions on the physical and sensory properties of T20. The addition of various amounts of water to the T20 formula is shown in Table 1. The appearance and color of T20 with various amounts of water added are shown in Figure 5. There was no significant difference in weight among the steamed breads (Table 8). However, the moisture content and water activity of T20 with various amounts of water added increased as the amount of water in the formula increased. The volume and specific volume of T20-15 and T20-20 were the highest among T20 samples, indicating that the formula of T20 with an extra 15–20% water added could improve the volume and specific volume of T20. This might be due to the fact that TP has higher total dietary fiber content (36.45%) and water holding capacity (2.97 g H_2_O absorbed/g sample) than wheat flour (Table 2), which enabled the steamed breads to retain more water vapor during steaming. However, when an extra 25–30% of water was added to the T20 formula, the texture structure of the sample became wet and sticky and collapsed, resulting in a decrease in product volume. The crust color, crumb color, aroma, flavor, texture, and overall preference scores of T20-15 and T20-20 were higher than those of other T20 samples, and there was no significant difference from the control (Table 8).

## 4. Conclusions

This study successfully developed a new formula of tamarillo steamed bread. Tamarillo is rich in dietary fiber and bioactive components and has good antioxidant capacity. Compared with traditional steamed breads prepared with 100% wheat flour, steamed breads prepared with up to 20% tamarillo powder partial replacement of wheat flour are rich in dietary fiber and bioactive components while having lower GI value and pleasant tamarillo flavor. Overall, tamarillo powder could be added to steamed breads to provide tamarillo steamed breads with more functional components and more effective antioxidant properties. Future studies will focus on an in vivo model to reflect the potential benefits of TP.

## Figures and Tables

**Table 1:** Formulae of tamarillo steamed breads.

Ingredient (%)		C ^1^	T5	T10	T15	T20	T20-05	T20-10	T20-15	T20-20	T20-25	T20-30
Wheat flour	middle protein flour	80	80	80	80	80	80	80	80	80	80	80
	low-protein flour	20	15	10	5	0	0	0	0	0	0	0
Tamarillo powder		0	5	10	15	20	20	20	20	20	20	20
Soybean powder		2	2	2	2	2	2	2	2	2	2	2
Yeast		2	2	2	2	2	2	2	2	2	2	2
Sugar		5	5	5	5	5	5	5	5	5	5	5
Shortening		1	1	1	1	1	1	1	1	1	1	1
Water		55	55	55	55	55	60	65	70	75	80	85
Total		165	165	165	165	165	170	175	180	185	190	195

^1^ Control, T5, T10, T15, and T20: steamed bread prepared with 0%, 5%, 10%, 15%, and 20% (*w*/*w*) replacement of low-protein flour with tamarillo powder, respectively. T20-05, T20-10, T20-15, T20-20, T20-25, and T20-30: formula of T20 added with extra 5%, 10%, 15%, 20%, 25%, and 30% (*w*/*w*) water added, with the amount of wheat flour in the control as 100%, respectively.

**Table 2:** Physicochemical quality characteristics of tamarillo powder and wheat flour.

	TP ^1^	MPF	LPF
Moisture (g/100 g)	1.28 ± 0.04 ^c2^	6.94 ± 0.09 ^a^	6.20 ± 0.09 ^b^
Crude fat (g/100 g)	0.94 ± 0.03 ^a^	0.93 ± 0.02 ^a^	0.94 ± 0.02 ^a^
Crude protein (g/100 g)	10.51 ± 0.21 ^b^	11.07 ± 0.08 ^a^	8.15 ± 0.08 ^c^
Crude ash (g/100 g)	6.91 ± 0.01 ^a^	0.38 ± 0.01 ^c^	0.44 ± 0.01 ^b^
Carbohydrate (g/100 g)	80.36 ± 0.21 ^c^	80.68 ± 0.18 ^b^	84.27 ± 0.02 ^a^
Total dietary fiber (g/100 g)	36.45 ± 9.21 ^a^	1.93 ± 0.73 ^b^	2.07 ± 0.89 ^b^
Water activity	0.254 ± 0.002 ^c^	0.641 ± 0.005 ^a^	0.593 ± 0.006 ^b^
Water holding capacity (g H_2_O absorbed/g sample)	2.97 ± 0.01 ^a^	0.81 ± 0.01 ^b^	0.59 ± 0.01 ^c^
Color property			
L*	53.50 ± 0.12 ^c^	93.11 ± 0.07 ^b^	93.98 ± 0.08 ^a^
a*	19.16 ± 0.12 ^a^	0.19 ± 0.04 ^b^	0.02 ± 0.03 ^b^
b*	21.91 ± 0.08 ^a^	8.61 ± 0.06 ^b^	7.26 ± 0.04 ^c^
WI*	44.99 ± 0.17 ^c^	88.97 ± 0.01 ^b^	90.57 ± 0.06 ^a^
c*	29.23 ± 0.12 ^a^	8.55 ± 0.06 ^b^	7.30 ± 0.04 ^c^
h* (*°*)	48.83 ± 0.01 ^c^	88.74 ± 0.01 ^b^	89.84 ± 0.01 ^a^
Δ*E*		45.99 ± 0.13 ^b^	47.23 ± 0.02 ^a^

^1^ TP: Tamarillo powder. MPF: Middle protein flour. LPF: Low-protein flour. ^2^ Each value is expressed as mean ± standard deviation (*n* = 3). Means with different lowercase letters within a row differ significantly (*p* < 0.05).

**Table 3:** The yield, bioactive components, and antioxidant property of tamarillo powder and wheat flour extracts.

	TP ^1^	MPF	LPF
Yield (g extract/100 g powder)	52.69 ± 0.86 ^a3^	7.16 ± 0.16 ^b^	6.31 ± 0.38 ^b^
Total phenols (mg GAE/g extract) ^1^	28.90 ± 0.20 ^a^	13.27 ± 0.24 ^b^	12.31 ± 0.51 ^c^
Ascorbic acid (mg/g extract)	3.25 ± 0.05 ^a^	0.32 ± 0.01 ^b^	0.31 ± 0.01 ^b^
Total anthocyanins (μg C3GE/g extract) ^1^	316.35 ± 0.02	nd ^4^	nd
Total carotenoids (μg βCE/g extract) ^1^	12.68 ± 1.33	nd	nd
EC_50_ value of antioxidant property (mg extract/mL) ^2^			
Scavenging ability of DPPH radicals	0.29 ± 0.01 ^c^	3.84 ± 0.05 ^b^	4.13 ± 0.02 ^a^
Reducing power	0.95 ± 0.05 ^b^	46.27 ± 0.32 ^a^	46.35 ± 0.72 ^a^

^1^ TP: Tamarillo powder. MPF: Middle protein flour. LPF: Low-protein flour. GAE: gallic acid equivalent. C3GE: cyanidin-3-glucoside equivalent. βCE: β-carotene equivalent. ^2^ EC_50_ value: The effective concentration at which DPPH radicals were scavenged by 50%, and the absorbance was 0.5 for reducing power. The EC_50_ value of TP was obtained by the interpolation method of linear regression analysis, while the EC_50_ value of flour was obtained by the extrapolation method of linear regression analysis. ^3^ Each value is expressed as mean ± standard deviation (*n* = 3). Means with different lowercase letters within a row differ significantly (*p* < 0.05). ^4^ nd: not detected.

**Table 4:** Physical quality characteristics of tamarillo steamed breads.

	Control	T5	T10	T15	T20
Weight (g)	25.25 ± 0.10 ^a1^	25.19 ± 0.19 ^a^	25.11 ± 0.10 ^a^	25.49 ± 0.34 ^a^	25.40 ± 0.11 ^a^
Dimension					
Length (mm)	59.15 ± 1.45 ^a^	58.90 ± 0.50 ^a^	45.90 ± 0.10 ^b^	43.20 ± 0.30 ^c^	43.70 ± 1.10 ^c^
Width (mm)	51.65 ± 0.65 ^a^	48.48 ± 0.60 ^b^	45.40 ± 0.30 ^c^	42.76 ± 0.25 ^d^	37.90 ± 0.6 ^e^
Height (mm)	32.65 ± 0.35 ^a^	32.11 ± 0.10 ^b^	31.25 ± 0.15 ^c^	31.05 ± 0.15 ^c^	28.75 ± 0.30 ^d^
Spread ratio	1.582 ± 0.004 ^a^	1.510 ± 0.034 ^b^	1.453 ± 0.023 ^c^	1.377 ± 0.021 ^d^	1.318 ± 0.023 ^e^
Volume (mL)	78.5 ± 0.7 ^a^	76.0 ± 0.1 ^b^	69.5 ± 0.7 ^c^	57.5 ± 0.7 ^d^	44.0 ± 1.4 ^e^
Specific volume (mL/g)	3.11 ± 0.03 ^a^	3.02 ± 0.02 ^a^	2.77 ± 0.02 ^b^	2.26 ± 0.05 ^c^	1.73 ± 0.05 ^d^
Crust color property ^2^					
L*	82.83 ± 0.49 ^a^	76.12 ± 1.30 ^b^	68.41 ± 0.79 ^c^	64.09 ± 0.62 ^d^	59.15 ± 0.66 ^e^
a*	−0.18 ± 0.08 ^e^	5.71 ± 0.14 ^d^	10.98 ± 0.25 ^c^	15.01 ± 0.44 ^b^	17.45 ± 0.59 ^a^
b*	11.15 ± 0.32 ^d^	25.77 ± 0.44 ^c^	34.18 ± 0.52 ^a^	34.95 ± 0.81 ^a^	31.36 ± 0.74 ^b^
c*	11.15 ± 0.32 ^d^	26.39 ± 0.43 ^c^	35.90 ± 0.48 ^b^	38.04 ± 0.75 ^a^	35.88 ± 0.88 ^b^
h* (*°*)	90.92 ± 0.26 ^a^	77.51 ± 0.20 ^b^	72.19 ± 0.26 ^c^	66.76 ± 0.67 ^d^	60.91 ± 0.29 ^e^
Δ*E*		17.12 ± 0.37 ^d^	29.38 ± 0.39 ^c^	33.89 ± 0.51 ^b^	35.77 ± 0.60 ^a^
Crumb color property					
L*	79.82 ± 1.08 ^a^	75.14 ± 0.86 ^b^	68.74 ± 0.98 ^c^	67.34 ± 0.79 ^d^	60.62 ± 0.74 ^e^
a*	−0.14 ± 0.01 ^e^	4.61 ± 0.15 ^d^	8.79 ± 0.17 ^c^	11.34 ± 0.19 ^b^	14.14 ± 0.40 ^a^
b*	12.17 ± 0.44 ^d^	24.62 ± 0.88 ^c^	30.83 ± 0.31 ^b^	31.94 ± 0.47 ^a^	30.77 ± 0.43 ^b^
c*	12.17 ± 0.44 ^d^	25.05 ± 0.88 ^c^	32.06 ± 0.31 ^b^	33.89 ± 0.47 ^a^	33.86 ± 0.47 ^a^
h* (*°*)	90.66 ± 0.16 ^a^	79.39 ± 0.26 ^b^	74.09 ± 0.11 ^c^	70.45 ± 0.31 ^d^	65.32 ± 0.21 ^e^
Δ*E*		14.12 ± 0.96 ^d^	23.47 ± 0.89 ^c^	26.14 ± 0.79 ^b^	30.33 ± 0.74 ^a^
Texture profile analysis					
Hardness (N)	4.86 ± 0.12 ^e^	6.18 ± 0.30 ^d^	7.61 ± 0.20 ^c^	10.08 ± 0.23 ^b^	11.65 ± 0.41 ^a^
Cohesiveness	0.81 ± 0.01 ^a^	0.74 ± 0.01 ^b^	0.69 ± 0.01 ^c^	0.64 ± 0.01 ^d^	0.61 ± 0.02 ^e^
Springiness	0.99 ± 0.02 ^a^	0.93 ± 0.02 ^a^	0.86 ± 0.03 ^b^	0.80 ± 0.04 ^c^	0.76 ± 0.05 ^c^
Gumminess (N)	3.94 ± 0.08 ^e^	4.57 ± 0.41 ^d^	5.25 ± 0.03 ^c^	6.45 ± 0.16 ^b^	7.11 ± 0.46 ^a^
Chewiness (N)	3.89 ± 0.11 ^e^	4.25 ± 0.37 ^d^	4.52 ± 0.14 ^c^	5.16 ± 0.35 ^b^	5.40 ± 1.44 ^a^
Resilience	0.43 ± 0.01 ^a^	0.29 ± 0.01 ^b^	0.27 ± 0.02 ^c^	0.23 ± 0.01 ^d^	0.21 ± 0.02 ^d^

^1^ Each value is expressed as mean ± standard deviation (*n* = 10). Means with different lowercase letters within a row differ significantly (*p* < 0.05). ^2^ L*, a*, b*, WI*, c*, h*, and Δ*E* values corresponding to lightness, redness, yellowness, whiteness index, chroma, hue angle, and total color difference, respectively.

**Table 5:** Proximate compositions, total dietary fiber, and water activity of tamarillo steamed breads.

	Control	T5	T10	T15	T20
Moisture (%)	37.58 ± 0.07 ^ab1^	37.66 ± 0.07 ^a^	37.48 ± 0.16 ^b^	37.25 ± 0.09 ^c^	37.02 ± 0.07 ^d^
Crude ash (%)	0.41 ± 0.01 ^e^	0.54 ± 0.01 ^d^	0.67 ± 0.03 ^c^	0.84 ± 0.01 ^b^	1.05 ± 0.01 ^a^
Crude fat (%)	0.61 ± 0.01 ^a^	0.61 ± 0.01 ^a^	0.61 ± 0.01 ^a^	0.62 ± 0.01 ^a^	0.62 ± 0.01 ^a^
Crude protein (%)	7.63 ± 0.04 ^c^	7.63 ± 0.05 ^c^	7.82 ± 0.11 ^b^	7.90 ± 0.17 ^b^	8.09 ± 0.04 ^a^
Carbohydrate (%)	53.77 ± 0.07 ^a^	53.56 ± 0.01 ^ab^	53.42 ± 0.171 ^b^	53.39 ± 0.184 ^b^	53.22 ± 0.07 ^b^
Total dietary fiber (%)	1.19 ± 0.47 ^e^	2.01 ± 0.53 ^d^	3.15 ± 0.091 c	4.25 ± 0.073 ^b^	5.11 ± 0.602 ^a^
Water activity	0.984 ± 0.004 ^a^	0.982 ± 0.062 ^a^	0.975 ± 0.058 ^a^	0.965 ± 0.053 ^b^	0.964 ± 0.058 ^b^

^1^ Each value is expressed as mean ± standard deviation (*n* = 3). Means with different lowercase letters within a row differ significantly (*p* < 0.05). Mean values of color and texture from 10 replicates, and mean values of other quality characteristics from three replicates.

**Table 6:** The yield, bioactive components, and antioxidant property of tamarillo steamed breads extracts.

	Control	T5	T10	T15	T20
Yield (g extract/100 g powder)	15.72 ± 0.11 ^e3^	18.48 ± 0.08 ^d^	20.63 ± 0.93 ^c^	24.09 ± 0.06 ^b^	25.43 ± 0.12 ^a^
Total phenols (mg GAE/g extract) ^1^	10.58 ± 0.25 ^e^	12.19 ± 0.54 ^d^	14.74 ± 0.05 ^c^	15.22 ± 0.08 ^b^	15.92 ± 0.06 ^a^
Ascorbic acid (mg/g extract)	0.241 ± 0.008 ^d^	0.384 ± 0.008 ^c^	0.546 ± 0.003 ^b^	0.621 ± 0.029 ^a^	0.627 ± 0.004 ^a^
Total anthocyanins (μg C3GE/g extract) ^1^	nd ^4^	243.1 ± 2.1 ^d^	265.5 ± 1.3 ^c^	357.1 ± 3.5 ^b^	389.3 ± 2.3 ^a^
Total carotenoids (μg βCE/g extract) ^1^	nd	25.39 ± 0.48 ^c^	27.25 ± 0.23 ^b^	27.14 ± 0.16 ^b^	30.34 ± 0.23 ^a^
EC_50_ value of antioxidant property (mg extract/mL) ^2^					
Scavenging ability of DPPH radicals	12.93 ± 0.06 ^a^	2.14 ± 0.10 ^b^	1.34 ± 0.12 ^b^	0.97 ± 0.04 ^b^	0.88 ± 0.05 ^b^
Reducing power	42.29 ± 0.38 ^a^	6.39 ± 0.21 ^b^	4.22 ± 0.18 ^c^	2.98 ± 0.72 ^d^	2.35 ± 0.04 ^e^

^1^ GAE: gallic acid equivalent. C3GE: cyanidin-3-glucoside equivalent. βCE: β-carotene equivalent. ^2^ EC_50_ values: The effective concentration at which DPPH radicals were scavenged by 50%, and the absorbance was 0.5 for reducing power. The EC_50_ values of tamarillo SB were obtained by the interpolation method of linear regression analysis, while the EC_50_ value of control was obtained by the extrapolation method of linear regression analysis. ^3^ Each value is expressed as mean ± standard deviation (*n* = 3). Means with different lowercase letters within a row differ significantly (*p* < 0.05). ^4^ nd: not detected.

**Table 7:** Predicted glycemic index (pGI) of tamarillo steamed breads in the in vitro test.

Sample	pGI	GI Category ^1^
Control	97.28 ± 0.47 ^A2^	High
T5	94.82 ± 0.38 ^B^	High
T10	89.72 ± 0.15 ^C^	High
T15	86.37 ± 0.44 ^D^	High
T20	80.02 ± 0.13 ^E^	Medium

^1^ GI categories, with the reference food of white bread are as follows: high GI value, GI > 85; medium GI value, 85 > GI > 60; and low GI value, GI < 60. ^2^ Each value is expressed as mean ± SD (*n* = 3). Means with different capital letters within a column differ significantly (*p* < 0.05).

**Table 8:** Physical quality characteristics and hedonic test results of control and T20 with various amounts of water added.

	Control	T20	T20-05	T20-10	T20-15	T20-20	T20-25	T20-30
Weight (g)	25.25 ± 0.10 ^a1^	25.40 ± 0.63 ^a^	25.25 ± 0.33 ^a^	24.95 ± 0.26 ^a^	25.31 ± 0.24 ^a^	25.15 ± 0.32 ^a^	25.29 ± 0.48 ^a^	25.06 ± 0.40 ^a^
Moisture (%)	38.72 ± 0.20 ^d^	38.46 ± 0.57 ^d^	39.49 ± 0.14 ^c^	40.10 ± 0.82 ^c^	43.18 ± 0.39 ^b^	43.81 ± 0.13 ^b^	45.46 ± 0.33 ^a^	45.67 ± 0.17 ^a^
Water activity	0.961 ± 0.006 ^d^	0.950 ± 0.003 ^e^	0.954 ± 0.001 ^e^	0.960 ± 0.001 ^d^	0.963 ± 0.003 ^cd^	0.967 ± 0.001 ^bc^	0.970 ± 0.001 ^ab^	0.972 ± 0.002 ^a^
Volume (mL)	78.50 ± 0.50 ^a^	44.00 ± 1.00 ^e^	62.77 ± 0.58 ^d^	65.33 ± 0.58 ^c^	70.67 ± 0.29 ^b^	68.63 ± 0.29 ^b^	65.50 ± 0.5 ^c^	64.00 ± 0.05 ^cd^
Specific volume (mL/g)	3.11 ± 0.03 ^a^	1.73 ± 0.05 ^d^	2.49 ± 0.06 ^c^	2.62 ± 0.02 ^bc^	2.79 ± 0.04 ^b^	2.73 ± 0.11 ^b^	2.59 ± 0.07 ^bc^	2.55 ± 0.06 ^bc^
Hedonic test								
Crust color	7.8 ± 1.1 ^a^	6.3 ± 1.3 ^c^	6.3 ± 1.3 ^c^	6.3 ± 1.3 ^c^	7.6 ± 1.2 ^a^	7.3 ± 1.1 ^ab^	6.8 ± 1.3 ^bc^	6.5 ± 1.2 ^c^
Crumb color	7.3 ± 1.3 ^a^	6.0 ± 1.2 ^b^	6.0 ± 1.4 ^b^	6.2 ± 0.8 ^b^	7.0 ± 1.5 ^a^	6.9 ± 1.5 ^a^	6.2 ± 0.9 ^b^	6.1 ± 1.1 ^b^
Sweetness	7.2 ± 1.6 ^a^	5.5 ± 1.2 ^c^	5.8 ± 0.9 ^bc^	5.9 ± 1.1 ^bc^	6.0 ± 1.0 ^bc^	6.0 ± 1.1 ^bc^	6.6 ± 1.5 ^abc^	6.8 ± 1.6 ^ab^
Aroma	7.0 ± 1.5 ^a^	5.4 ± 1.2 ^b^	5.5 ± 1.2 ^b^	6.5 ± 1.0 ^a^	6.8 ± 1.4 ^a^	6.9 ± 1.3 ^a^	6.4 ± 1.0 ^a^	6.5 ± 1.1 ^a^
Flavor	7.1 ± 1.3 ^a^	5.4 ± 1.2 ^c^	5.7 ± 0.8 ^c^	5.9 ± 0.9 ^c^	6.9 ± 1.0 ^a^	6.9 ± 1.5 ^a^	6.8 ± 1.4 ^ab^	6.1 ± 1.3 ^bc^
Texture	7.6 ± 0.9 ^a^	5.4 ± 1.1 ^b^	6.3 ± 0.5 ^b^	6.6 ± 1.4 ^b^	7.6 ± 1.2 ^a^	7.8 ± 1.0 ^a^	7.8 ± 1.1 ^a^	7.6 ± 1.4 ^a^
Overall	7.6 ± 0.9 ^a^	5.6 ± 0.8 ^e^	6.8 ± 1.2 ^cd^	6.9 ± 0.9 ^bcd^	7.5 ± 0.7 ^ab^	7.4 ± 1.1 ^abc^	6.7 ± 1.2 ^d^	6.5 ± 1.0 ^d^

^1^ Means ± standard deviation (*n* = 90 for hedonic scale test and *n* = 3 for others) with different lowercase letters within a row differ significantly (*p* < 0.05). Nine-point hedonic scale with 1, 5, and 9 representing extremely disliked, neither liked nor disliked and extremely liked, respectively.

## Data Availability

Data are contained within the article.
